# Exendin-4 protects mice from D-galactose-induced hepatic and pancreatic dysfunction

**DOI:** 10.1080/20010001.2017.1418593

**Published:** 2017-12-26

**Authors:** Akram Ahangarpour, Ali Akbar Oroojan, Mohammad Badavi

**Affiliations:** ^a^ Diabetes Research Center, Department of Physiology, Health Research Institute, Ahvaz Jundishapur University of Medical Sciences, Ahvaz, Iran; ^b^ Department of Physiology, Student Research Committee of Ahvaz Jundishapur University of Medical Science, Ahvaz, Iran; ^c^ Physiology Research Center, Department of Physiology, Ahvaz Jundishapur University of Medical Sciences, Ahvaz, Iran

**Keywords:** Metabolic dysfunction, D-galactose, exendin-4, antioxidant enzymes, aging

## Abstract

Investigations into pharmaceutical intervention of pancreatic and hepatic dysfunction associated with metabolic disturbances have received relatively little attention. The aim of this study was to investigate the protective effects of exendin-4 in mice receiving D-galactose, a reducing sugar that triggers ROS production and inflammatory mediators affecting the pancreas and liver. Exendin-4 is an United States Food and Drug Administration (FDA) approved glucagon-like peptide that increases insulin dependent glycogen synthesis and glucose uptake. Male NMRI mice (20–25 g), 3 months of age, were randomly divided into 6 groups of 12 mice each: control, exendin-4 (1 nmol/kg), exendin-4 (10 nmol/kg), D-galactose, D-galactose + exendin-4 (1 nmol/kg) and D-galactose + exendin-4 (10 nmol/kg). D-galactose (500 mg/kg) was given daily by oral gavage for 6 weeks. During the last 10 days, exendin-4 (1 and 10 nmol/kg) was injected intraperitoneally daily. Glucose, insulin, insulin resistance, lipid profiles, and hepatic enzyme levels significantly increased in the D-galactose group (*p* < 0.05), along with a significant decrease in superoxide dismutase activity and pancreatic islet insulin secretion (*p* < 0.05). Exendin-4 decreased D-galactose-induced increases in serum glucose and insulin, insulin resistance, lipid profiles, and hepatic enzymes, and improved pancreatic islet insulin secretion and antioxidant defense status. The results show that exendin-4 can prevent complications in mice with compromised pancreatic and hepatic function. Long term administration of D-galactose in mice may be a useful model to study insulin resistance, metabolic syndrome, and aging.

## Introduction

Metabolic syndrome is associated with aging, obesity, oxidative stress, insulin resistance, increased blood glucose, impaired glucose tolerance, increased insulin, decreased HDL, increased triglycerides, and increased LDL [1]. Villegas et al. showed a close relationship between elevated liver aminotransferases (ALT and AST) and metabolic syndrome; an increase in these enzyme levels is a primary risk factor for chronic diseases associated with aging [2]. Two of the major organs involved in the pathogenesis of metabolic dysfunction are pancreas and liver. A number of drugs are being studied for treatment and prevention of metabolic syndrome, but investigations into pharmaceutical intervention of pancreatic and hepatic function associated with metabolic disturbances have received less attention. Exendin-4 is an United States Food and Drug Administration (FDA) approved glucagon-like peptide-1 that increases insulin-dependent glycogen synthesis and the liver’s glucose uptake [3]. In one study, exendin-4 demonstrated a reduction in ROS generation and an enhancement effect on antioxidant enzyme activity, such as SOD, GPX, and CAT in rats [4]. Exendin-4 has been used for glucose stimulated insulin secretion, gastric emptying, and appetite suppression in type 2 diabetic patients. When administered, exendin-4 reduced liver lipids, plasma alanine transaminases, cholesterol, and triglycerides in both humans and mice. Exendin-4 can also facilitate insulin secretion in ß-cells through glucose sensor activation, such as glucokinase [5]. Recently, exendin-4 showed a lipid accumulation reduction and improved hepatic lipid metabolism in mice fed a high fat diet. Further, exendin-4 attenuates hepatic lipogenesis via ß-catenin activation [6]. Exendin-4 has insulinotropic, antidiabetic, and glucoregulatory effects through the pancreatic GLP-1 receptor. This drug also has a stimulatory action on glucose metabolism in the liver [7].

The present study was designed to explore the antioxidant and protective effects of exendin-4 in mice with compromised pancreatic and hepatic function. The model selected was daily administration of D-galactose, a reducing sugar which can be converted into aldose and hydro peroxide, resulting in anion superoxide and ROS production, such as hydrogen peroxide and superoxide radicals [8]. Systemic administration of D-galactose triggers ROS formation in mitochondria and enhances advanced glycation end (AGE) products through activation of their receptor RAGE. Over-expressed RAGE activates NF-kB and other inflammatory mediators that may be associated with metabolic disorders, aging and age-related diseases, especially affecting the pancreas and liver [9].

## Materials and methods

### Animals and treatment design

Three month old male Naval Medical Research Institute (NMRI) mice each weighing between 20–25 g, were purchased from Ahvaz Jundishapur University of Medical Sciences (AJUMS) animal facility. Animals used in this study were treated in accordance with AJUMS’ principles and guidelines for animal care with ethical number D-9209 and were kept at between 20°C and 24°C under a 12 h light/dark cycle with free access to tap water and food (commercial mouse chow: Pars Animal Feed Co., Tehran, Iran). Mice were randomly divided into six groups (*n* = 12 in each group): control, exendin-4 (1 nmol/kg), exendin-4 (10 nmol/kg), D-galactose, D-galactose + exendin-4 (1 nmol/kg), D-galactose + exendin-4 (10 nmol/kg). D-galactose (500 mg/kg) (Merck, Germany) was dissolved in drinking water, and given by oral gavage daily for 6 weeks [10]. Control groups were orally gavaged drinking water with the same volume without D-galactose. Exendin-4 (1 and 10 nmol/kg) (Sigma, USA) was dissolved in phosphate buffered saline (PBS) (Merck, Germany) and injected intraperitoneally daily during the last 10 days of D-galactose administration [11,12]. Control groups received PBS only. Twenty-four hours after the last drug administration, 1.5 ml of blood were collected from overnight-fasted control and treated animals under anesthesia by ketamine/xylazine (70 and 10 mg/kg, respectively, in combination) [13] using cardiac puncture. Serum was harvested from blood samples for clinical pathology.

### Pancreatic islet collection

Pancreatic islets were isolated as described [14] and transferred to Petri dishes containing Krebs-bicarbonate buffer with basal (2.8 mM), moderated (5.6 mM) and stimulated (16.7 mM) glucose concentrations [15]. All samples were then shaken by vortex and incubated at 4°C for 24 h followed by incubation in a 37°C water bath for 30 min. The samples were transferred to 2 mL microtubes and centrifuged at 100 × *g* for 5 min by refrigerated centrifuge; 0.9 mL of supernatant were collected and maintained at −70°C until the insulin secretion assays were performed. Each Petri dish contained seven islets, and the procedure was repeated eight times for each animal in all groups [14].

### Clinical pathology assessment

Serum glucose was measured using biochemical assay kits (Pars Azmoon, Iran). Insulin levels of serum and islet secretion samples were evaluated using ELISA assays kits (Monobind, USA) (The sensitivity of hormone detection per assay tube was 0.182 µIU/ml). HOMA-IR (homeostasis model assessment-estimated insulin resistance) was calculated according to the following formula: fasting insulin (µIU/dL) × fasting glucose (mg/dL)/405 [16]. Lipid profile and hepatic enzymes in serum samples were measured using biochemical assay kits (Pars Azmoon, Iran) and an Autoanalyzer device (BT3000, Italy).

### Antioxidant assessment

Serum antioxidant enzyme SOD (ZB-SOD-96A), GPX (ZB-GPX-96A), and CAT (ZB-CAT-96A) activities were measured using ELISA assays kits (ZellBio GmbH, Germany), according to the provider’s instructions.

### Statistical analysis

Data were statistically analyzed using SPSS software with one-way analysis of variance (ANOVA) and least significant difference (LSD) tests. The results were expressed as mean ± SEM (standard error of means) and differences were considered statistically significant at *p* < 0.05.

## Results and discussion

**Figure 1.**
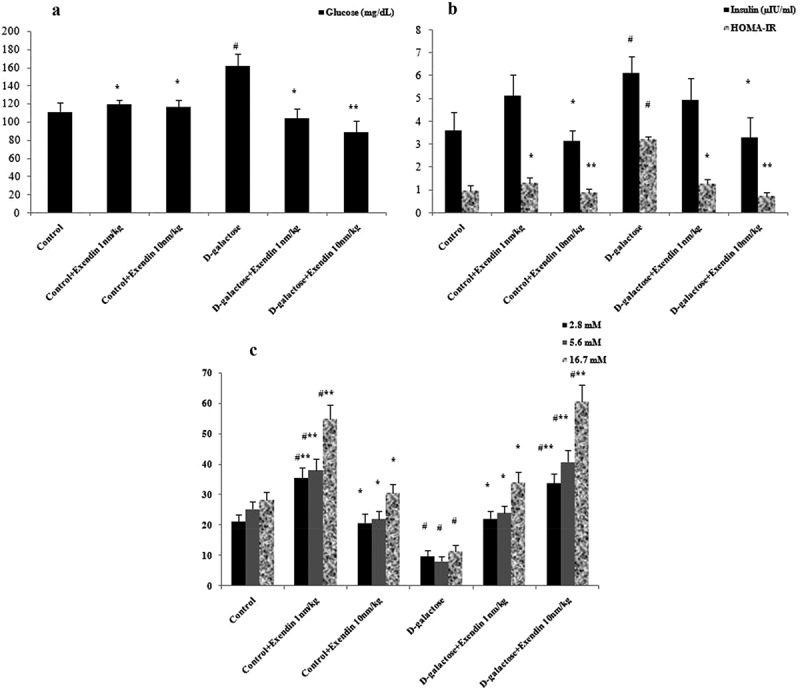
Treatment with exendin-4 in NMRI mice decreased serum glucose (a), insulin and HOMA-IR (b), and increased insulin secretion by pancreatic islets cultured in medium containing different concentrations of glucose (c). Data are expressed as the mean ± SEM of 12 mice in each group for glucose, insulin and, HOMA-IR, and 8 samples for islet insulin secretion. Data were analyzed using one-way analysis of variance and least significant difference tests. * *p* < 0.05 and ** *p* < 0.01 designate significant differences with D-galactose treatment group, ^#^ *p* < 0.05 designates significant differences with the control group.

Exendin-4 attenuated D-galactose-induced hyperglycemia, hyperinsulinemia and insulin resistance (Figure 1(a,b)). Glucose increased in the D-galactose treated mice in comparison to D-galactose + exendin-4 (10 nmol/kg) (*p* < 0.01) and other groups (*p* < 0.05). An increase was evident in the serum insulin level in the D-galactose group when compared with exendin-4 (10 nmol/kg) and D-galactose + exendin-4 (10 nmol/kg) groups (*p* < 0.05). HOMA-IR showed an increase in the D-galactose group compared with exendin-4 (10 nmol/kg), D-galactose + exendin-4 (10 nmol/kg) (*p* < 0.01), and other groups (*p* < 0.05). Insulin secretion from isolated islets was decreased in the D-galactose versus control group (*p* < 0.05) (Figure 1(c)). Administration of exendin-4 (1 nmol/kg) increased this secretion in normal animals when compared with the control (*p* < 0.05) and D-galactose (*p* < 0.01) groups. Further, exendin-4 at 10 nmol/kg improved islet insulin secretion in treated mice when compared with the D-galactose (*p* < 0.01) and control (*p* < 0.05) groups. This secretion was increased in exendin-4 (10 nmol/kg) and D-galactose + exendin-4 (1 nmol/kg) groups versus the D-galactose group (*p* < 0.05). Moreover, insulin secretions occurred in a similar manner in all medium concentrations of glucose (2.8, 5.6 and 16.7 mM). One of the effects of exendin-4 is an increase in glucokinase enzyme activity in the liver through a parallel and independent insulin-mediated mechanism [3]. It has been shown that glucokinase can initiate phosphorylation of glucose after it has diffused into liver cells. Glucose is temporarily trapped inside the liver cells because phosphorylated glucose cannot diffuse back through the cell membrane [17]. These observations suggest that lowering of serum glucose by exendin-4 in the present study may have been achieved by a similar mechanism.

**Figure 2.**
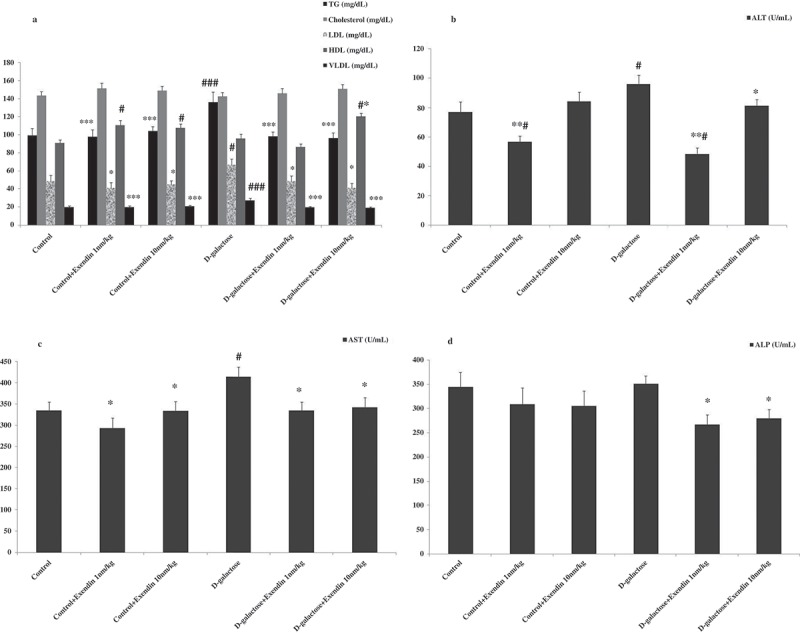
Treatment of NMRI mice with exendin-4 altered hepatic lipid and enzymatic profiles in the serum. (a) Serum TG, LDL, and VLDL levels were decreased while serum HDL was increased. (b) ALT, (c) AST, and (d) ALP were all decreased in the serum of mice treated with exendin-4. Data are expressed as the mean ± SEM of the 12 mice in each group. Data were analyzed using one-way analysis of variance and least significant difference tests. * *p* < 0.05, ** *p* < 0.01 and, *** *p* < 0.001 designate significant differences with D-galactose treatment group, ^#^ *p* < 0.05 and ^###^ *p* < 0.001 designate significant differences with control group.

The results of lipid profiles show that D-galactose did increase serum levels of triglyceride (TG), low density lipoprotein (LDL), and very-low-density lipoprotein (VLDL), and that treatment with exendin-4 decreased this profile (Figure 2(a)). No difference was observed in serum cholesterol levels among groups. Interestingly, administration of this drug resulted in an increase in the serum level of HDL in normal and D-galactose treated mice. TG and VLDL showed increases when compared with other groups (*p* < 0.001). LDL in D-galactose treated mice increased in comparison with other groups (*p* < 0.05). Serum HDL levels in normal animals that received exendin-4 (1 and 10 nmol/kg) showed an increase versus the control group (*p* < 0.05). This lipid factor in the D-galactose + exendin-4 (10 nmol/kg) treated group increased when compared with the D-galactose group (*p* < 0.05). Exendin-4 as a GLP-1 receptor agonist can decrease LDL, apolipoprotein B, TG, and increase HDL. Research by Parlevliet et al. [18] indicated that this drug could reduce hepatic steatosis and ameliorate dyslipidemia by decreasing serum levels of VLDL and TG. The corresponding results are in agreement with the present study showing that exendin-4 can reduce harmful lipid factors and increase the serum level of HDL.

Increases were seen in serum levels of alanine transaminase (ALT) in the D-galactose treated group when compared with the control group and the D-galactose + exendin-4 (10 nmol/kg) (*p* < 0.05), exendin-4 (1 nmol/kg) and D-galactose + exendin-4 (1 nmol/kg) treated groups (*p* < 0.01) (Figure 2(b)). Further, aspartate aminotransferase (AST) increased in D-galactose treated mice when compared with other groups (*p* < 0.05) (Figure 2(c)). Exendin-4 (1 and 10 nmol/kg) decreased the alkaline phosphatase (ALP) contents in serum samples of D-galactose treated mice (*p* < 0.05) (Figure 2(d)). The liver is an essential organ that performs a wide range of biochemical, metabolic, and drug metabolites [19]. AST, ALT, and ALP enzymes are liver biomarkers associated with liver dysfunction or damage [20]. Exendin-4 has been shown to improve hepatic function by decreasing aminotransferase levels and increasing hepatic antioxidants in rats fed a high fat diet. The protective and curative effects of this drug on hepatic steatosis may occur through regulation of redox and reduction of inflammation. The underlying mechanisms of these effects are associated with RAGE expression in the liver stellate and Kupffer cells that lead to elevated uptake of AGEs [21].

SOD enzyme activity decreased in mice that received D-galactose in comparison with other groups (*p* < 0.01) (Table 1). Exendin-4 (1 nmol/kg) increased this enzyme activity in normal mice and D-galactose-treated mice when compared with control groups (*p* < 0.05 and *p* < 0.01). This effect was observed in the D-galactose + exendin-4 (10 nmol/kg) group when compared with the control group (*p* < 0.05). Exendin-4 (10 nmol/kg) increased CAT enzyme activity, but not GPX activity, in normal mice (*p* < 0.05) as well as in the D-galactose + exendin-4 (10 nmol/kg) group versus the D-galactose group (*p* < 0.05) (Table 1). Increased SOD and CAT enzyme activities by exendin-4 in normal and D-galactose treated animals is in agreement with a study by Gezginci-Oktayoglu et al. [22] showing that the administration of exendin-4 for 30 days improved antioxidant defenses. CAT is a primary antioxidant enzyme that converts H2O2 to water, so if CAT activity decreases in cells, it may lead to the accumulation of H2O2 and cause DNA damage or cell death [23]. Higher levels of SOD activity can actually produce higher levels of H2O2, which is normally detoxified by CAT. Thus, exendin-4 improves D-galactose induced dysfunction through the enhancement of both SOD and CAT enzyme activity most likely as the result of a decline in free radicals (anion superoxide).

**Table 1.** Exendin-4 increased serum antioxidant enzyme activity in both D-galactose treated and control-treated mice.

Group	Treatment	SOD (U/ml)	CAT (U/ml)	GPX (U/ml)
D-galactose	Placebo	0.55 ± 0.09^##^	6.07 ± 0.74	394.28 ± 22.14
	Exendin-4 1 nM	1.84 ± 0.3**	5.8 ± 0.91	344.21 ± 21.62
	Exendin-4 10 nM	2.62 ± 0.22^#,^**	8.91 ± 1.15*	408 ± 26.74
Control	Placebo	1.59 ± 0.25	7.45 ± 0.96	394.73 ± 28.22
	Exendin-4 1 nM	2.43 ± 0.29^#,^**	7.41 ± 0.35	384.63 ± 17.67
	Exendin-4 10 nM	1.97 ± 0.26**	10.74 ± 0.94^#^	407.36 ± 26.64

Data are expressed as the mean ± SEM using 12 mice per group. Data were analyzed using one-way analysis of variance and least significant difference tests. *p < 0.05 and **p < 0.01 designate significant differences with D-galactose placebo treatment group, ^#^ *p* < 0.05 and ^##^ *p* < 0.01 designate significant differences with control placebo group. SOD: superoxide dismutase; CAT: catalase; GPX: glutathione peroxidase.

There is considerable evidence that free radicals play an important role in the development of insulin resistance, impaired glucose tolerance, pancreatic islet cell dysfunction, and type 2 diabetes [24]. Insulin secretion by isolated pancreatic islets revealed that the administration of D-galactose can induce ß-cell dysfunction and decrease secretion possibly by ROS generation. The fact that exendin-4 treatment was able to attenuate D-galactose-induced reduction of insulin secretion suggests that exendin-4 can alter oxidant signaling. The observations by Kaneto et al [25] showed that catalase and SOD can protect ß-cells against ROS-induced damage. Also, antioxidant treatment can suppress apoptosis of ß-cells and preserve ß-cell function in diabetic mice. Exendin-4 can promote glucose-stimulated insulin gene transcription, biosynthesis, secretion, and pancreatic ß-cell mass in rodents and in type2 diabetic patients. These events compensate for peripheral insulin resistance [26]. Therefore, results of the present study suggest that exendin-4 can ameliorate D-galactose-induced ß-cell dysfunction through an increase in antioxidant enzyme activities.
